# An automated framework to classify skin lesions using Multi-Head Self Attention Layer-based Vision Transformers

**DOI:** 10.3389/frai.2026.1781796

**Published:** 2026-05-05

**Authors:** Sahil Faizal, Charu Anant Rajput, Manas Ranjan Prusty

**Affiliations:** 1 School of Computer Science and Engineering, Vellore Institute of Technology, Chennai, India; 2 Centre for Cyber Physical Systems, Vellore Institute of Technology, Chennai, India

**Keywords:** contrast stretching, Multi-Head Self Attention Layer, multi-layer perceptron, skin lesion detection, Vision Transformers

## Abstract

Skin lesions are one of the most prevalent form of diseases existing among us. Early detection and classification of potentially malignant skin lesions can give us a lead in the fight against skin cancer. There are many lesion classification divisions on medical grounds; however, an automated system that detects and classifies a majority of these classes is not prevalent. In view of this scenario, our proposed study aims to classify the input skin lesion images into nine classes, namely, squamous cell carcinoma (SCC), Basal cell carcinoma (BCC), melanocytic nevi (NV), actinic keratoses and intraepithelial carcinoma (AKIEC), melanoma (MEL), seborrheic keratosis (SEK), dermatofibroma (DF), benign keratosis like lesions (BKL), and vascular lesions (VASC). The proposed methodology uses contrast stretching as an image enhancement technique to facilitate efficient Region of Interest (ROI) segmentation. The novelty of the proposed study lies in the first-hand implementation of Vision Transformer (ViT) for feature extraction in the domain of skin lesion detection. Finally, a light-weight multi-layer perceptron (MLP) composed of fully connected layers is used for multinomial classification. Combining the aforementioned techniques, the proposed method achieves training accuracy of 98% and testing accuracy of about 93.22%. The impressive performance across nine distinct categories represents a significant milestone. This success demonstrates the model’s scalability, suggesting it can be effectively extended to a broader array of diagnostic classes in future research.

## 1 Introduction

Skin lesions are abnormal areas of the skin with an abnormal appearance or tissue growth that may be painful or itchy. The appearance typically differs from the surrounding skin. Dermatologically, these lesions can be primary or secondary, in which primary lesions appear naturally as a defect at birth or develop over time owing to environmental factors. Secondary lesions that originate from primary lesions are often more critical.

There are seven primary classes in which skin lesions have been segregated, according to the majority of publicly available datasets. As described by Shetty et al. (2022) actinic keratoses and intraepithelial carcinoma (AKIEC) are non-invasive growths of squamous cells that can be treated without the need for surgical intervention. Basal cell carcinoma (BCC) develops at the epithelial level and rarely spreads, but can be fatal if left untreated. Benign keratosis like lesions (BKL) are primarily benign in nature and appear as brown, black, or light tan on the skin. Dermatofibroma (DF) appears on the dermis layer and is typically benign in nature as well. However, melanoma (MEL) is the most dangerous, as it is malignant and arises from the formation of cancerous cells in melanocytes (skin-color-giving cells). Melanocytic nevi (NV) are benign in nature and appear as abnormally dark patches on the skin surface. Vascular lesions (VASC) can be benign or malignant and are typically formed in underlying tissues or organs. These are the main classes as mentioned in the HAM10000 dataset.

Apart from the aforementioned classes, PAD-UFES also has two additional classes called squamous cell carcinoma (SCC), whose localization and spread are on the squamous cells, which make up the middle and outer layers of the skin. In contrast, seborrheic keratosis (SEK) is again benign in nature and generally develops with age and is a kind of epidermal tumor. Diagnosis becomes particularly difficult owing to the variety of skin lesion formations and the presence of minute, subtle differences in their appearance. Medically, an invasive technique involving sample collection from the affected region is performed, and the presence of malignancy is confirmed by microscopic and histopathological examination. However, it is not always possible to perform such a practice owing to the sensitivity of the afflicted region and, therefore, in such scenarios, determining the diagnosis using appearance appears to be the key method.

Therefore, a variety of studies involving deep learning and medical image processing in view of the aforementioned scenario have been developed. Shetty et al. (2022) have proposed a comparison between the six state-of-the-art machine learning (ML) models (Decision Tree, Random Forest, Support Vector Machine [SVM], k-nearest neighbor [KNN], Linear Regression, Gaussian Naïve Bayes, and Linear Discriminant Analysis [LDA]) and the convolutional neural network (CNN) architecture Inception-V3. The study makes use of the HAM10000 dataset, wherein the images have been resized to a dimension of 220 px × 220 px, and features like texture, color, and shape have been extracted to come out with the final classification results. The performance of the CNN, with training accuracy up to 95.18% and testing accuracy up to 86.43%, clearly surpassed that of the ML models, which were barely achieving 50%. Similarly, Lee et al. (2019) make use of the segmentation architecture wherein DenseNet has been used as an encoder, and U-Net has been used as a decoder and presented as an ensemble model. Voting based on a weighted score is used to determine the classification results. The proposed study makes use of the International Skin Imaging Collaboration (ISIC) 2018 challenge dataset and produces a training accuracy of 89.9% and validation accuracy of 78.5%. Di̇Mi̇Li̇Ler and Sekeroglu (2022) have used the PAD-UFES architecture, in which the implementation is based on a CNN architecture. Here, convolutional and pooling layers have been used for feature extraction, whereas dense layers have been used for classification. The six classes are grouped into skin disease and skin cancer, each containing three classes, and the Rectified Linear Unit (ReLU) has been used as the activation function. The accuracy for the binomial classification is 86%, whereas that for the multinomial classification is 86.5%. Hameed et al. (2020) have proposed an approach in which feature extraction is performed across three color formats: Hue, Saturation, Value (HSV), Luma, Chroma Blue, Chroma Red (YCbCr), and Grayscale. These are then fed into Multi Class Single Level and Multi Class Multi Level models, thereby generating classification results in terms of healthy, eczema, benign, and malignant, with an accuracy of 96.4%.

Recently, apart from deep learning and image augmentation techniques, transformers have been used to enhance the performance of computer-vision-based medical diagnosis. It has been named as Vision Transformer (ViT) and forms the crux of our proposed study. The following survey points out some of the applications of ViT across different fields, highlighting our motivation and idea behind incorporating its usage in skin lesion detection. Bazi et al. (2021) have proposed using a Vision Transformer to classify the different view fields in an image. It has been tested on four datasets: Merced, Aerial Image Dataset (AID), Optimal31, and the Northwestern Polytechnical University (NWPU) dataset. Yamabe and Saitoh (2022) conducted a comparative study between CNN and ViT for bark image recognition, and concluded that ViT significantly outperformed CNN, achieving 96.57 and 96.30% accuracy on the NewBarkTex and Trunk12 datasets, respectively. Chen et al. (2021) in their study shed light on how ViT can be made to perform across image patches of varying sizes by generating separate processing branches on the basis of the size of the tokens and then applying a cross-attention-based token fusion technique for generating complementary-sized tokens for classification purposes. In case of perturbed tokens during image capture, Li et al. (2023) proposed an entirely new model, Bootstrap Own Latent Transformer (BOLT), to accurately perform classification. It has found applications across diverse medical fields like skin lesion classification, knee fatigue, fracture grading, and diabetic retinopathy. Accuracy figures of up to 81.5% for the ISIC dataset have been registered. Dai et al. (2021) combined the strengths of CNNs and Transformers for feature extraction on multimodal images in the Parotid Gland Tumor (PGT) and MRNet datasets, achieving 88.9 and 94.9%accuracy, respectively.

Apart from this, there are research studies extensively involving the use of transfer learning and ensemble learning for the purpose of achieving the required goal of skin lesion classification. Hosny et al. (2019) have made use of a fine-tuned AlexNet model by tweaking the training weights, introducing a softmax layer for making the classifications, and additionally using dataset augmentation. The proposed fine-tuned deep learning model is implemented over three publicly available datasets, namely, MED-NODE, Derm (IS & Quest), and ISIC, thereby producing an accuracy of 96.86, 97.70, and 95.91%, respectively. Ratul et al. (2020) have proposed the use of dilated convolution to improve the model’s performance against blockers such as surrounding-skin resemblance and fuzzy lesion borders. The technique has been implemented using transfer learning across four prominent models, namely, VGG16, VGG19, MobileNet, and Inception-V3, achieving accuracies of 87.42, 85.02, 88.22, and 89.81%, respectively. Rehman et al. (2022) have proposed an approach wherein two deep learning models, namely, MobileNetV2 and DenseNet201, are pipelined together by putting three convolutional layers in between. This modified version of the DenseNet201 model achieves an accuracy of 95.50% when applied to the ISIC dataset. Gessert et al. (2018) have proposed the formation of a pipeline model combining pre-trained state-of-the-art models such as DenseNet, SENet, and ResNeXt. Additionally, the novelty of the work lies in the use of unscaled crops to validate the proposed model. The accuracy thus produced after processing over the HAM10000 dataset is 85%. Mirunalini et al. (2017) have made use of fine-tuned Inception V3 for the purpose of achieving the task of skin lesion detection over the ISIC dataset.

Skin lesions require higher contrast for accurate Region of Interest (ROI) segmentation and identification. Contrast stretching is a kind of enhancement technique wherein the distribution of digital numbers across multiple pixels is modified individually. Afza et al. (2022) used Contrast Street Enhancement to enhance the lesion region and subsequently demonstrated the application of hybrid whale optimization and an entropy mutual function for feature selection. Feature fusion is done using canonical correlation, and extreme machine learning has been used to generate classification results. HAM10000 and ISIC2018 have been used, and accuracy figures of up to 93.40 and 94.36% have been reported. Firdausy et al. (2007) have deep-dived into exploring the technique of contrast stretching and concluded that Gamma Correction, a histogram-based technique based on a tuning factor called gamma, is the preferred approach. Negi and Bhandari (2014) have presented Contrast Enhancement in conjunction with Image Sharpening. The proposed study also highlights contrast stretching with adaptive thresholding for MRI knee images and concludes with satisfactory results. Malik et al. (2022) have proposed a novel technique called Hybrid Metaheuristic Differential Evolution-Bat Algorithm to enhance lesion contrast while preserving the contrast stretching transformation function. Baig et al. (2019) have proposed an approach for implementing local and global contrast stretching to classify cancerous and non-cancerous cells, treating the image sequentially under edge detection, color detection, and subsequent segmentation. This is followed by the extraction of statistical features to serve as the classification criteria. Nugroho et al. (2019) used a convolutional neural network framework to classify the HAM10k dataset, producing training and testing accuracies of 80 and 78%, respectively. Alwakid et al. (2022) used the same HAM10k dataset with the proposed CNN-based model, achieving an accuracy of 0.86. This method used a modified version of Resnet-50 and CNN for melanoma detection. Popescu et al. (2022) used the collective intelligence of multiple neural networks for the same classification task on the HAM10k dataset. Extreme gradient booting-based deep learning model (Khan et al., 2021), lightweight HierAttn (Dai et al., 2022), and Metadata supervision (Pundhir et al., 2022) are a few of the other techniques that have been used for skin lesion classification. Pandey et al. (2024) present a multi-stage approach for skin disease detection that combines image processing techniques, non-local means denoising for targeted ROI extraction, and a convolutional neural network (CNN) supported by sparse dictionary learning. However, the achieved accuracy 85.61%. Agrawal et al. (2024) present a lightweight deep CNN model for the diagnosis of skin lesions. To address the class imbalance issue, the approach employs an adaptive class-balanced focal loss function, which enhances the model’s learning capability by immediately diverting effort over hard-to-classify examples. The model achieves an overall accuracy of 91.88%. De et al. (2024) present a hybrid model for detecting skin conditions utilizing DenseNet and ResNet. Methods including hybridization, batch normalization, and data fitting are used in the model optimization process to improve the architecture and data handling. To improve learning from informative images, Iraji (2025) present a weighted bad semi-supervised generative adversarial network (WB-SGAN) that combines a weighted inverse cross-entropy loss function with self-social consistency regularization. Pham et al. (2025) present a deep ensemble framework that combines three distinct convolutional neural network (CNN) architectures, each equipped with efficient attention mechanisms. However, the framework achieves an accuracy of only 84.35%. As witnessed, a majority of the published studies in the domain of skin lesion classification are dominated by the use of CNN and ensemble learning. This is where the proposed study aims to bring and broaden the perspective by inculcating the use of Transformers in the domain of skin lesion classification and identification.

Recent reviews of transformer-based dermatological implementations reveal a consistent pattern: accuracy tends to decrease as the number of diagnostic classes increases, especially when attempting to minimize the complexity of the processing architecture. Himel et al. (2024) attempt to make a binomial classification between malignant and benign skin lesions by employing the use of the Segment Anything Model (SAM) for explicit ROI segmentation coupled with ViT for feature extraction and a classifier, producing an accuracy of 97.12%. Similarly, Jenefa et al. (2024) also employ the use of ViT with a multi-layer perceptron (MLP) head in their model called DEEPSCAN for the purpose of performing binomial classification, but the main differentiator in this case is the additional use of patient metadata and histopathological imaging. Hybrid CNN (ResNet-50 first 10 layers) with Vision Transformer encoder developed by Nie et al. (2022) produces an accuracy of 89.48% for seven classes, whereas for the same number of classes, CNN-based transfer learning with ViT developed by Arshed et al. (2023) produce an accuracy of 92.14%. Additionally, Vachmanus et al. (2023) attempted to perform multi-modal classification considering seven to eight diagnostic classes under scope by employing the use of Bidirectional Encoder Representation from Image Transformers (BEiT) and a combination of datasets like ISIC 2018, PAD-UFES-20, and Fitzpatrick17k, which could propose an accuracy of 87.1%. Zhang et al. (2023) have developed a custom multi-modal transformer with a mid-point transverse process to pleura (MTP) block that classifies five classes, achieving an accuracy of 77.99%. While Khan and Khan (2023) perform a trinomial classification between melanoma and nonmelanoma (basal cell carcinoma [BCC] and squamous cell carcinoma [SCC]) with an accuracy of 91.02%.

Section 2 presents a high-level overview of the proposed methodology, and Section 3 deals with the proposed methodology followed during the image preprocessing phase, which includes a description of up-sampling and the application of contrast stretching enhancement. Section 4 presents a detailed description of the proposed model architecture. Section 5 presents the resultant facts and figures in terms of the confusion matrix and other evaluation metrics. Section 7 concludes the overall study with references for future enhancements.

## 2 Proposed methodology

**Figure 1:**
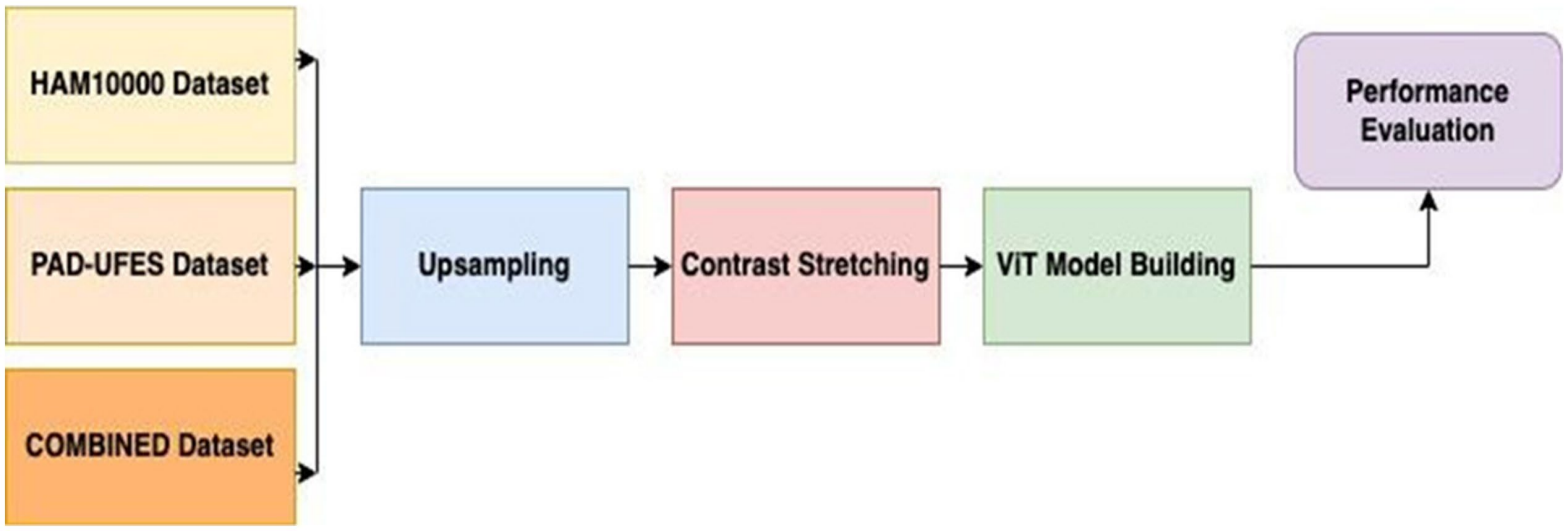
High-level workflow of the proposed approach for skin lesion classification.

The proposed study aims to present the implementation of the model for 3 types of multinomial classification: nine classes, six classes, and seven classes. A combination of HAM10000 (Tschandl et al., 2018) and PAD-UFES (Pacheco et al., 2020) has been used to produce nine classes, and the study also presents the performance figures for HAM10000 and PAD-UFES individually. Image preprocessing involved up-sampling the images belonging to each class to a size of 1,000 using various image augmentations, followed by the key enhancement technique called contrast stretching. The proposed model employs a Vision Transformer (ViT) for feature extraction, followed by a multi-layer perceptron (MLP) composed of fully connected layers for final classification. Figure 1 represents the high-level workflow of the proposed methodology. Initially, the HAM10k and PAD-UFES datasets are combined, and then they undergo the up-sampling procedure using different augmentation techniques, such as zoom, flip top, flip bottom, random brightness, and distortion. Contrast stretching enhancement (CSE) is used for efficient ROI segmentation. This is followed by feature extraction using Vision Transformers (ViT), and finally, classification is performed based on the inherently vectorized, non-spatial features thus extracted.

The proposed model and all associated code trials have been run in the Google Collab Pro environment with a high-performance Graphics Processing Unit (GPU), Nvidia A100, and the availability of high in-memory instances (up to 32 GB of processing RAM). TensorFlow and Keras have been imported to leverage their powerful built-in libraries and associated functionalities for image augmentation and MLP model formation. Python is the primary programming language utilized throughout the project lifecycle, encompassing data extraction from dataset metadata, model development, and the generation of performance evaluation reports.

### 2.1 Dataset collection

**Figure 2:**
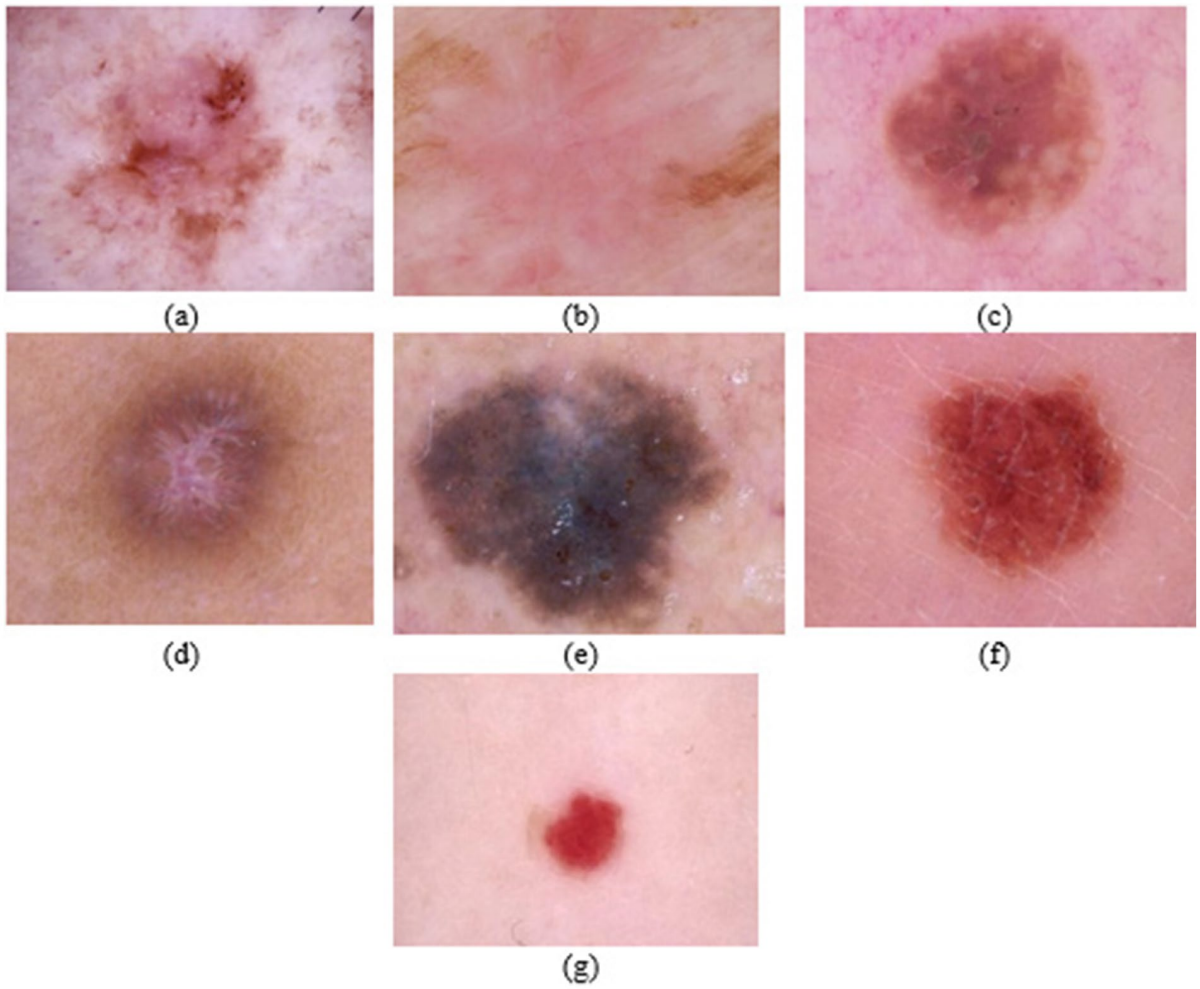
**(a)** Actinic keratosis and intraepithelial carcinoma (AKIEC/Bowen’s disease); **(b)** Basal cell carcinoma (BCC); **(c)** Benign keratosis like lesions (BKL); **(d)** Dermatofibroma (DF); **(e)** Melanoma (MEL); **(f)** Melanocytic nevi (NV); **(g)** Vascular lesions (VASC).

**Figure 3:**
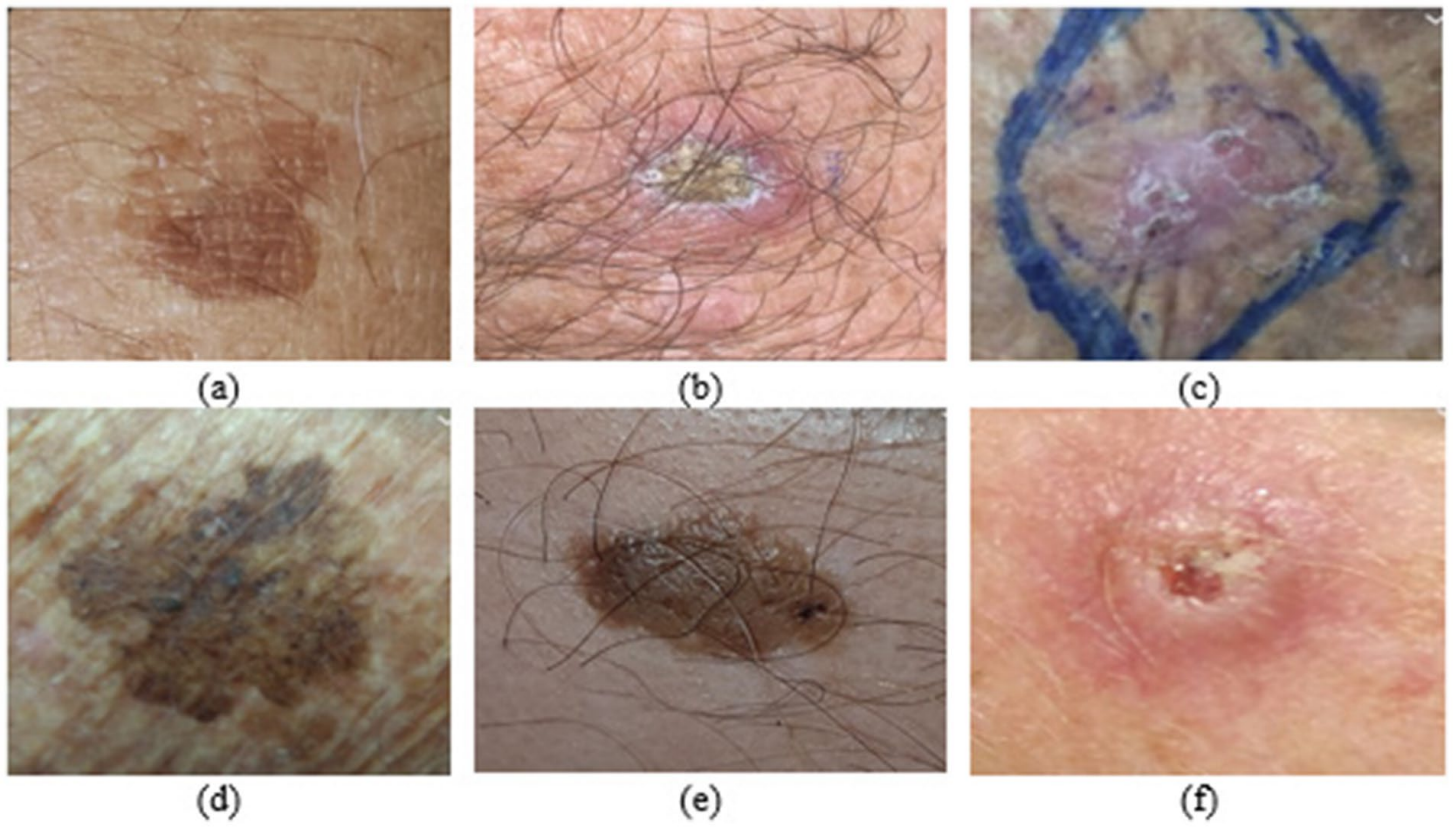
**(a)** Seborrheic keratosis (SEK); **(b)** Actinic keratosis (ACK); **(c)** Basal cell carcinoma (BCC); **(d)** Melanoma (MEL); **(e)** Nevus (NEV); **(f)** Squamous cell carcinoma (SCC).

The HAM10000 dataset is a collection of 10,000 images distributed across seven classes: Actinic keratoses and intraepithelial carcinoma (AKIEC), basal cell carcinoma (BCC), benign keratosis (BKL), dermatofibroma (DF), melanoma (MEL), melanocytic nevi (NV), and vascular lesions (VASC). The dataset was available in two parts, each part containing 5,000 and 5,015 images. The image format was jpeg. However, the images were not perfectly segmented into designated classes in each part, and the process had to be performed using a Python script over the metadata csv file. As opposed to HAM10000, which is medically curated by the Department of Dermatology at the Medical University of Vienna, the images of PAD-UFES are captured using smartphones, contain data from about 1,373 patients with 1,641 lesions, and comprise a total of 2,298 images across six diagnostic classes. Three of them have been categorized as skin diseases, and the other three as skin cancers. Finally, to obtain the nine-class classification, we combined the HAM10000 and PAD-UFES classes while up-sampling each to reach a total of 1,000. It is to be duly noted that the train-test split was performed before the up-sampling process, which was used to ensure that the input candidate images for the classes had the same count. Table 1 gives a glimpse of the numeric distribution of images in the aforementioned datasets. According to the process, the classes with fewer than 1,000 images have been up-sampled appropriately, whereas 1,000 or more images have been selected randomly Finally, all these operations lead to the formation of nine classes, each with 1,000 images, resulting in the formation of 9 classes, each with 1,000 images, resulting in a combined dataset containing 9,000 images. Figures 2, 3 refer to the images of diverse disease classes represented from the HAM10k dataset and PAD-UFES datasets, respectively. From the following tables, it can be inferred that some common classes are present, which makes it possible to combine the datasets.

**Table 1:** Image class distribution across datasets.

Type	PAD-UFES	SKIN-HAM10000
Actinic keratosis and intraepithelial carcinoma (AKIEC)	730	327
Melanocytic nevi (NV)	244	6,705
Basal cell carcinoma (BCC)	845	514
Squamous cell carcinoma (SCC)	192	0
Melanoma (MEL)	52	1,113
Seborrheic keratosis (SEK)	235	0
Benign keratosis like lesions (BKL)	0	1,099
Vascular lesions (VASC)	0	142
Dermatofibroma (DF)	0	115

## 3 Image preprocessing

The image preprocessing stage primarily involves contrast stretching. Following the initial train-test split, the augmentation process was applied using various operations, including zoom, vertical and horizontal flips, distortions, and random brightness adjustments. All these operations have been performed using the Keras and Augmentor packages. By executing these operations in specific ratios, the image count for each disease category, previously standardized to 1,000, was increased to nine distinct classes, each containing 2,000 images, providing a comprehensive training set of 18,000 images for the training process.

The contrast stretching technique is used to enhance image contrast locally. The reference for the aforementioned technique is discussed in Afza et al. (2022), which describes a hybrid contrast stretching technique based on absolute mean deviation and skewness functions. The aforementioned technique is useful for efficiently segmenting the lesions from the background skin area and also removes unwanted noise.

**Figure 4:**
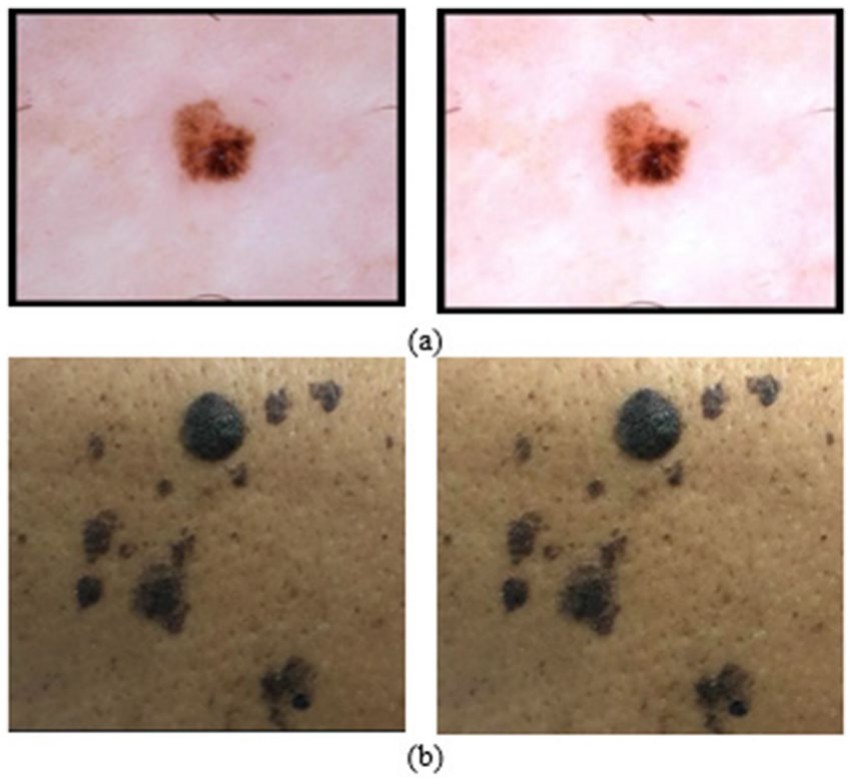
**(a)** Implementation of contrast stretching on HAM10k dataset; **(b)** Implementation of contrast stretching on PAD-UFES dataset.

Afza et al. (2022) have presented the mathematical interpretation of contrast stretching. Considering the input image X of dimension N × M, wherein n is the total number of pixels, and x_i_ is each image pixel. The formulations of absolute mean deviation and skewness are represented in Equations 1, 2. Figures 4a,b represents the implementation of contrast stretching on the original input images.


$$\tilde{\mathrm{MD}}=\frac{1}{n}\sum_{i=1}^{n}|x_{i}-\underline{\emptyset }(X)|$$ (1)

$$\tilde{\mathrm{SK}}=\frac{\sum_{i=1}^{n}{\left(x_{i}-\underline{X}\right)}^{3}}{(n-1)\times \sigma^{3}}$$ (2)

## 4 Model architecture

Transformers have traditionally been used for the purpose of Natural Language Processing based applications, in which the input sentence is subdivided into tokens and, after semantic and syntactic analysis, the underlying intent is identified. Even the famous ChatGPT applications are based on Generative Pre-trained Transformer (GPT) to identify and model the dependencies between words in an input text. However, transformers are not widely used in the domain of Computer Vision, and the majority of research works rely heavily on CNN-based architectures, primarily ResNet, VGG-16, Inception V3, ensemble-based learning models, and fine-tuned architectures that employ transfer learning. Vision Transformers is one of the first approaches to incorporate a transformer encoder in the domain of computer vision and can surpass CNN performance while reducing computational resource consumption by 4×. The ViT model essentially represents an input image as a series of patches and classifies it based on the categorization of these patches. This is quite analogous to the Language Modeling approach, which is used for tasks concerning future token prediction.

This transformer is composed of the following three main components:

(i) Multi-Head Self Attention (MHSA) Layer: Responsible for computing the attention across multiple parallel self-heads, wherein each head in turn captures different aspects of feature relationships within the given input image. The outputs from all the heads are finally concatenated and passed through a linear projection to generate a unified, better refined feature representation.
(ii) Multi-layer perceptron: It has two fully connected (FC) layers with Gaussian Error Linear Unit (GELU) sandwiched between these two layers. GELU is responsible for introducing non-linearity, which boosts the model’s ability to recognize complex patterns within the input image.
(iii) Layer norm (LN): Used for optimizing the performance model by preventing the occurrence of overfitting and is added prior to each of the processing blocks. This is instrumental in improving training convergence and thereby contributes to improving overall model performance.

Self-attention and Attention maps are two such features that allow Vision Transformers to successfully segment non-essential regions of the input image, such as healthy skin and surface hairs. Self-attention allows the model to capture different regions of the input data by computing weighted sums based on the similarity of input features. In this way, it helps the processing model capture and focus on information-rich segments of the image. Attention maps, in contrast, are matrices that demonstrate the importance of different parts of the image and can be visualized as a grid of heat maps showing the attention weights between a given token and all the other tokens. Corresponding to the number of Transformer layers, we employ 12 attention maps. The brighter colored pixels represent higher attention weight and, therefore, can be separated as a part of ROI segmentation.

### 4.1 Proposed model architecture

According to the proposed approach, the Vision Transformer has been adopted to use as the primary feature extractor due to its computational efficiency and optimized resource consumption. The implementation involves the use of a multitude of packages like glob and os for file handling, cv2 for handling the operations about computer vision, numpy for handling numerical computations and setting the dimensions of the input image, TensorFlow and Keras for running the model and performing image preprocessing tasks like image augmentation and subsequent test-train split. Finally, matplotlib is used to plot the relevant accuracy and loss curves.

The input images have been resized to 224 × 224 for the purpose of reducing computational intensity. This is followed by converting each of these images into numpy arrays and normalizing the pixel values by dividing each by 255. This is further followed by applying contrast stretching enhancement as the primary operation under the image preprocessing phase. The input data is further split into training and test sets at an 80:20 ratio. Note that the images in the training split now undergo the augmentation and up-sampling.

**Figure 5:**
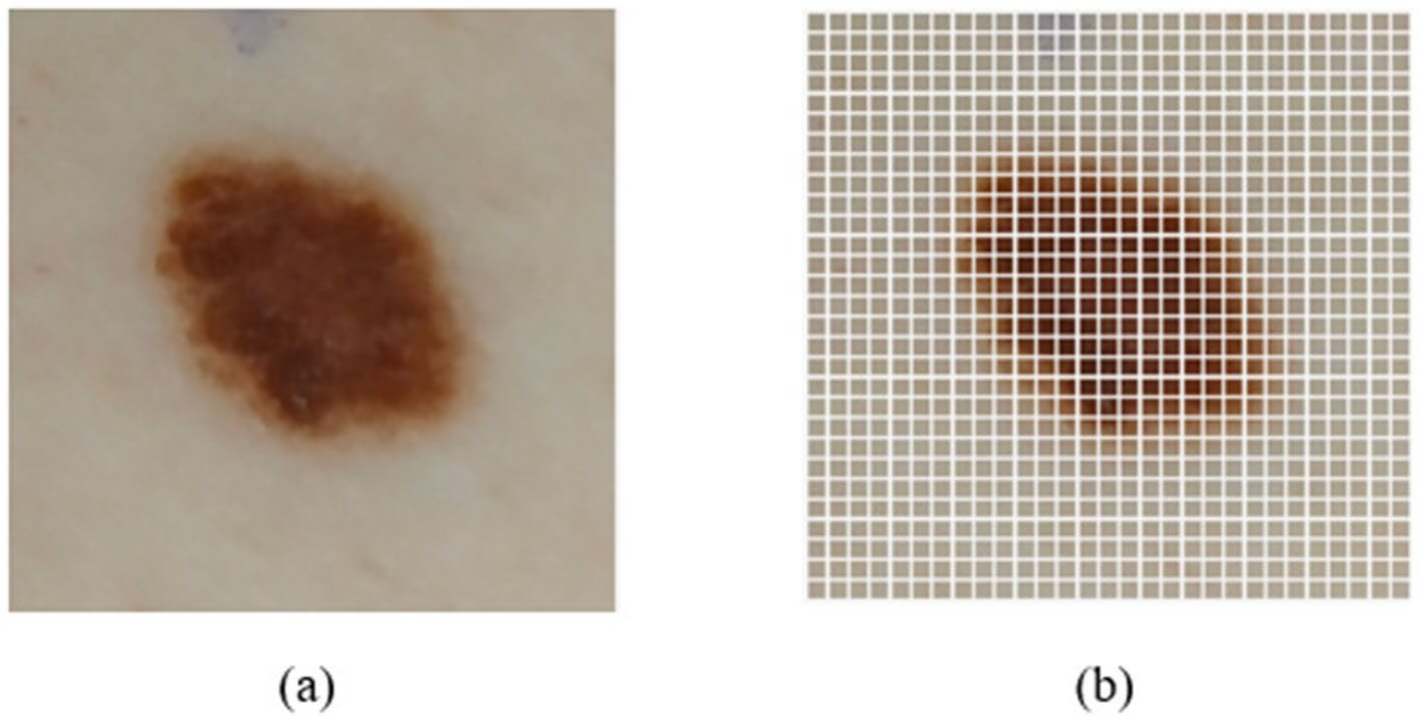
Implementation of ViT on an input skin patch image. **(a)** Original Input Image; **(b)** Image after implementing ViT.

This is followed by importing the ViT model from the ViT Keras package (specifically vit-base-patch32-384), which is then initialized. This model specifically employs 12 transformer layers. The ViT model has been pre-trained using ImageNet weights. Additionally, we use the Adam optimizer with a learning rate of 0.0001 and categorical cross-entropy as the loss function. Figure 5 represents the implementation of ViT onto an input image. An input skin image of dimension 224 × 224 is divided into 49 non-overlapping patches, each of dimension 32 × 32, containing 3,072 raw pixel values (32 × 32 × 3 channels). Each patch is linearly projected into a 768-dimensional embedding, to which a positional embedding is added to retain spatial information.

**Figure 6:**
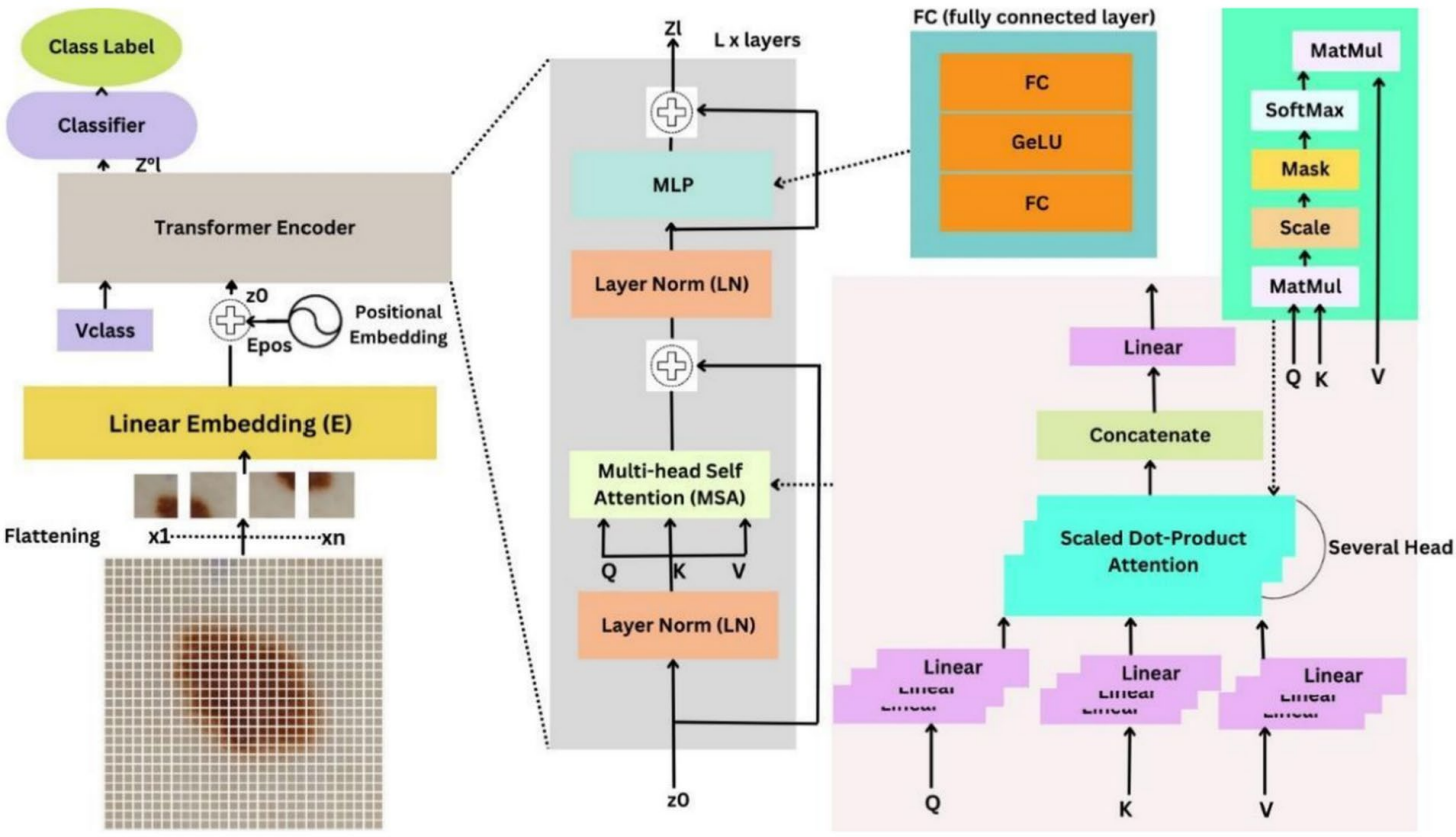
Schematic diagram of the proposed Multi-Head Self-Attention Enabled Vision Transformer Architecture (MSA-ViT).

Figure 6 presents the nuances of the proposed architecture. Generally, the architecture consists of an embedding layer, an encoding layer, and a final head classifier, as described in Bazi et al. (2021). The input image X of dimensions *c* × *h* × *w* (where *h* is the height, *w* is the width, and *c* represents the number of channels, which in our case equals 224, 224, and 3, respectively). From this input image, patches of dimensions *c* × *p* × *p* are extracted, where p is the patch size and is 32 in our case. This leads to the formation of a sequence of patches of length n, where *n = hw/p^2^.* These patch sequences are further fed into the linear embedding layer without any regard to order. The Linear Embedding layer essentially feeds the patch sequence as a model vector of dimension d to the encoder. As mentioned in the aforementioned section, the encoder is composed of three units: LN, MHSA, and MLP. The output thus produced by the encoder is finally fed to the external head classifier, which can be any ML algorithm or a custom-made fully connected layer for predicting the corresponding class label. The classification head consists of a flattened layer followed by fully connected (dense) layers and dropout for regularization. The ReLU activation function introduces non-linearity, while kernel regularization and batch normalization are used to mitigate overfitting and enhance training stability. The training process has been run for 20 epochs, with an input batch size of 32.

## 5 Results and discussion

This section presents the proposed model’s performance against the aforementioned performance measures. Apart from this, the section presents results from 5-fold cross-validation and the receiver operating characteristic (ROC) curve. Section 5.1 reports the model’s performance on a combination of the HAM10k and PAD-UFES datasets. Sections 5.2 and 5.3 deal with the model’s performance on the PAD-UFES and HAM10k datasets, respectively.

### 5.1 Combined data (HAM10K + PAD-UFES)

**Figure 7:**
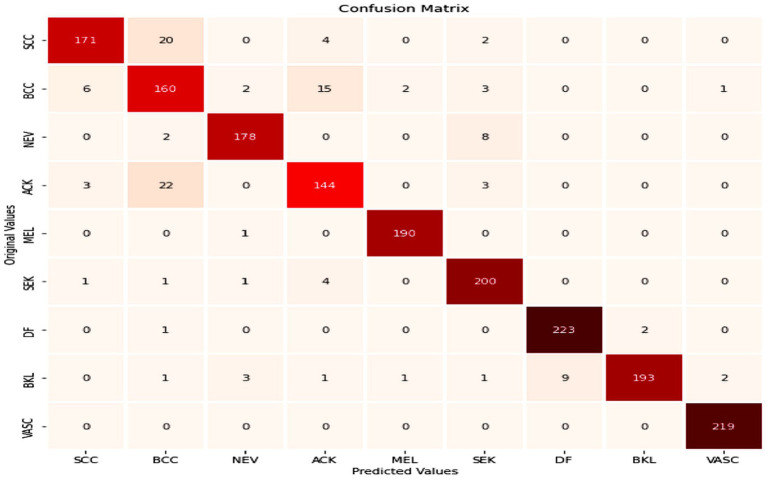
Confusion matrix for performance on the combined dataset.

**Figure 8:**
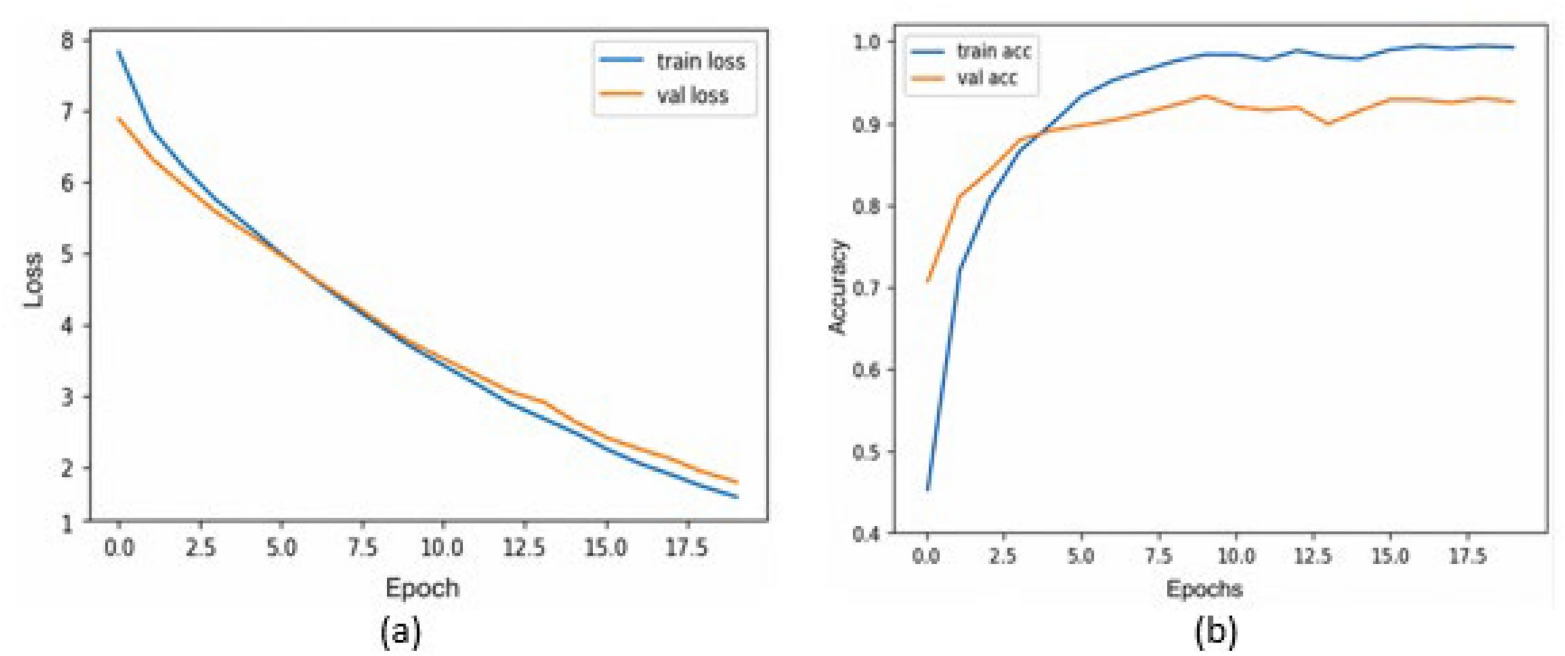
**(a)** Training loss and validation loss vs. number of epochs; **(b)** Training accuracy and Validation accuracy vs. the number of epochs.

**Figure 9:**
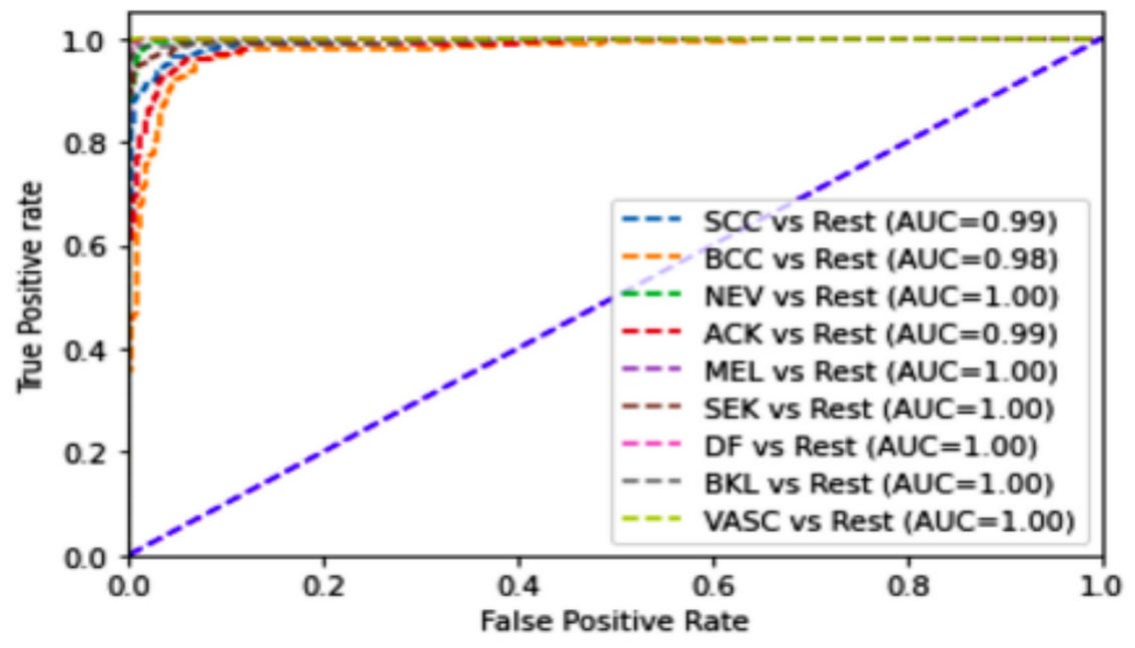
Multiclass ROC curve for (HAM10k + PAD-UFES) dataset.

Table 2 presents the performance of the proposed model on a combination of the HAM10k and PAD-UFES datasets, as measured by the performance metrics. It also displays the metrics from the 5-fold cross-validation. The consistency between the 5-fold cross-validation and hold-out validation results demonstrates the model’s robustness and its ability to generalize well to unseen data. It is to be noted that, since nine classes participate as class labels in the proposed study, the measures of precision, recall, f1-score, and specificity have been averaged and presented as a single figure demonstrating the overall performance of the proposed model. The confusion matrix for the same is shown in Figure 7, where the diagonal entries represent the number of correct predictions, and the rows correspond to the count of images in the validation dataset for each class. From the confusion matrix, it can be concluded that the disease categories, MEL, DF, and SEK, were the top three most accurately classified. Additionally, Figures 8a,b represents a comparison between the loss and accuracy of the training and validation datasets, respectively. The graphs show how the model’s accuracy or loss of behavior changed over the course of training and as the number of epochs increased. Table 3 represents the model’s performance in predicting each class against standard performance metrics. Apart from this, the performance of the model has been further represented by means of receiver operating characteristic (ROC) curve which signifies the model’s ability to detect the ability to distinguish between different classes. ROC and area under the ROC curve (AUC) are applicable for models concerning binary classification. To meet the requirements of multinomial classification, we employ the One vs. Rest (OvR) strategy. According to this, each class is compared against the rest, wherein the class under evaluation is considered positive, and the rest are negative. Figure 9 shows the multiclass ROC curve. AUC value obtained from Figure 9 is 0.99410. The AUC value ranges from 0 to 1, and the closer it is to 1, the better its performance is considered. Similarly, the ROC curve shows greater perpendicularity when the AUC approaches 1. All the aforementioned validation methods have been performed using the validation (testing) dataset.

**Table 2:** Overall performance of the model on (HAM10k + PAD-UFES) dataset.

Category	Accuracy (%)	Precision (%)	Recall (%)	Specificity (%)	F1-score (%)
Hold-out cross-validation	93.22	93.4134	93.223	93.145	93.2544
5-Fold cross-validation	93.199	93.42	93.10	93.21	93.24

**Table 3:** Class-wise performance metrics on (HAM10k + PAD-UFES) dataset.

Class	Precision	Recall	F1-score	Support	Accuracy
SCC	94	87	90	197	86.80
BCC	77	85	81	189	87.43
NEV	96	95	95	188	94.68
ACK	86	84	85	172	85.20
MEL	98	99	99	191	99.47
SEK	92	97	94	207	96.61
DF	96	99	97	226	98.67
BKL	99	91	95	211	91.47
VASC	99	100	99	219	100

### 5.2 PAD-UFES dataset

**Figure 10:**
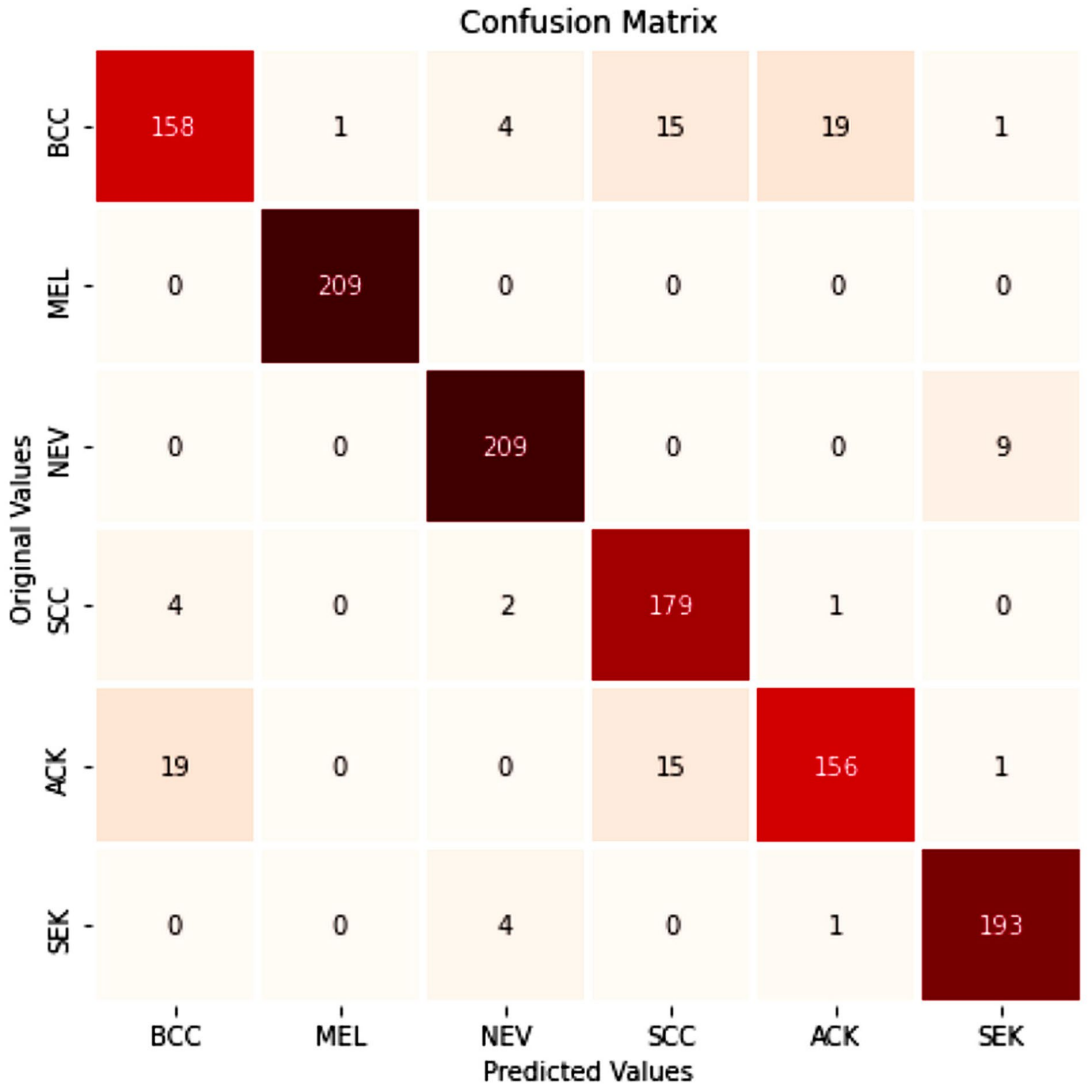
Confusion matrix for performance on PAD-UFES dataset.

**Figure 11:**
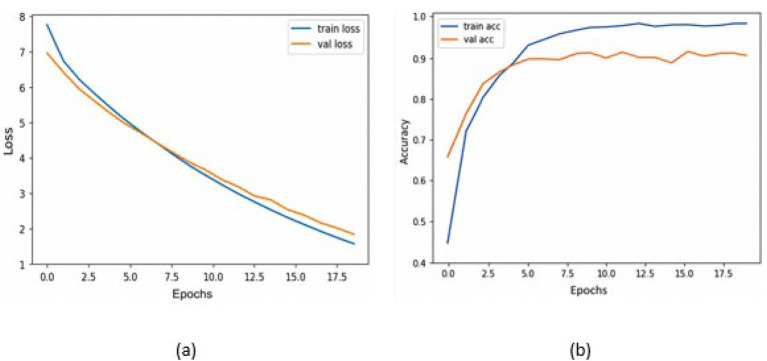
**(a)** Training loss and validation loss vs. number of epochs; **(b)** Training accuracy and validation accuracy vs. the number of epochs.

**Figure 12:**
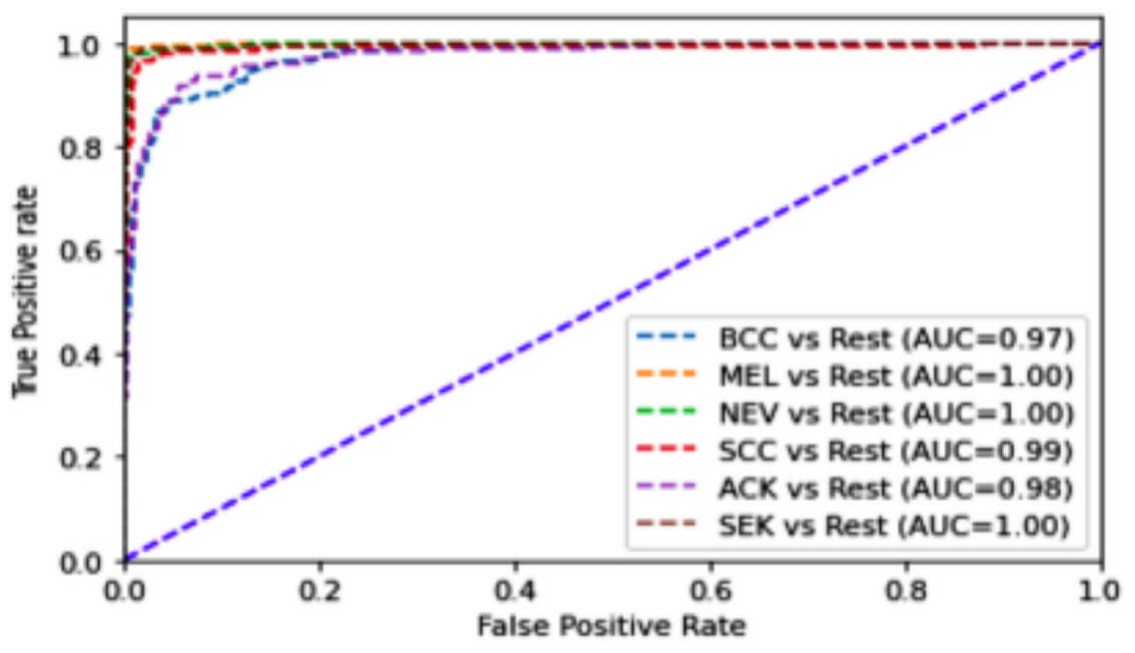
Multiclass ROC curve for PAD-UFES dataset.

Table 4 presents the model’s performance on the PAD-UFES dataset using standard performance metrics. Figure 10 shows the confusion matrix for evaluating the model’s performance on the PAD-UFES dataset. Figures 11a,b shows the loss and accuracy curves for the PAD-UFES dataset, respectively. Table 5 shows the model’s ability to accurately identify the classes individually on the PAD-UFES dataset. Figure 12 shows the corresponding ROC curve. AUC value obtained from the ROC curve shown in Figure 12 is 0.9889.

**Table 4:** Overall model performance on PAD-UFES dataset.

Category	Accuracy (%)	Precision (%)	Recall (%)	Specificity (%)	F1-score (%)
Hold-out cross-validation	92.15	92.185	92.16	92.11	92.08
5-Fold cross-validation	92.10	92	92.15	92.099	92.08

**Table 5:** Class-wise performance metrics on the PAD-UFES dataset.

Class	Precision	Recall	F1-score	Support	Accuracy
BCC	82	85	84	196	79.79
MEL	100	98	99	218	100
NEV	94	98	96	216	95.87
SCC	93	95	94	195	96.23
ACK	90	77	83	185	81.67
SEK	93	98	95	190	97.47

### 5.3 HAM10k dataset

**Figure 13:**
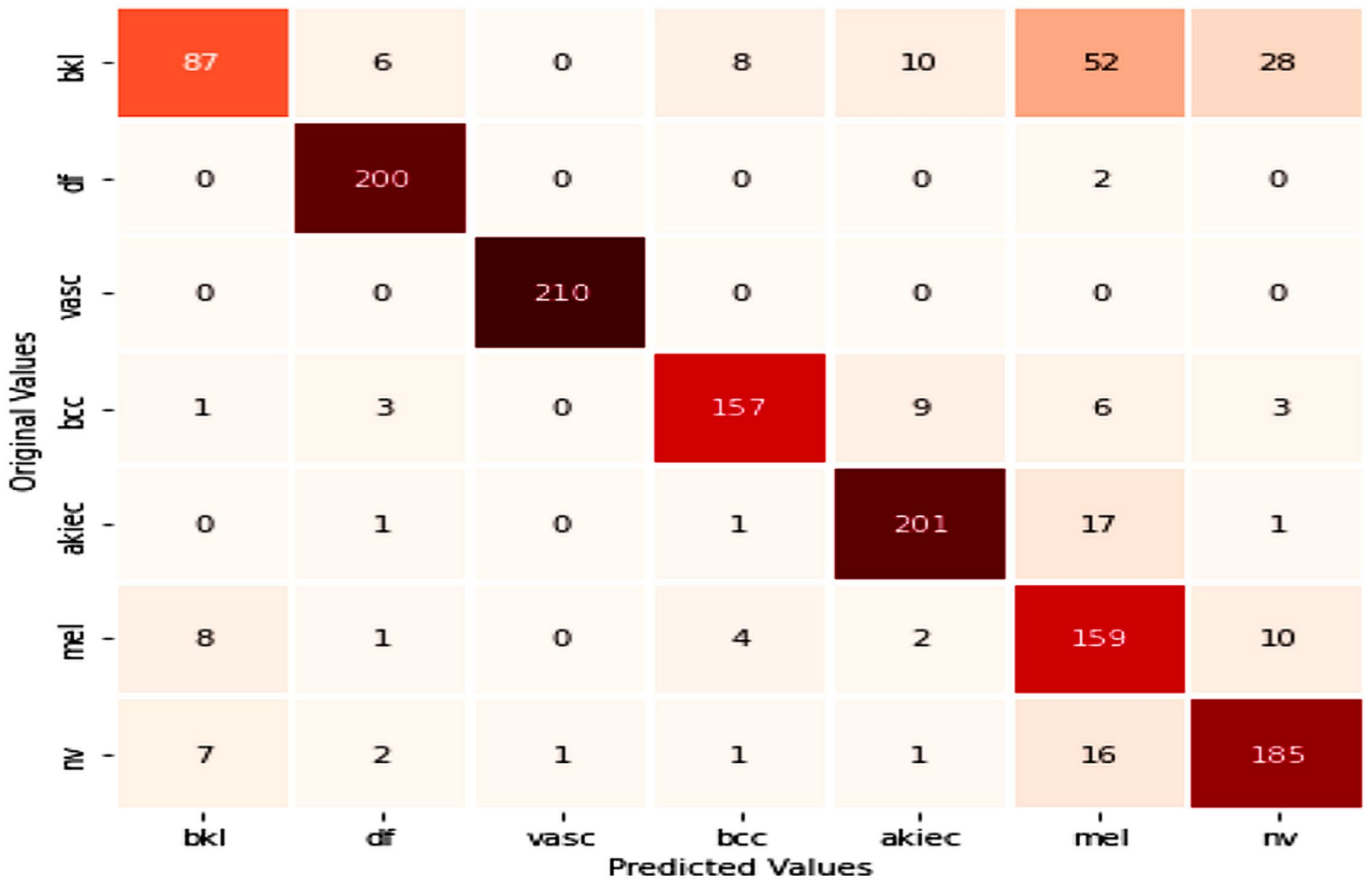
Confusion matrix for performance on HAM10k dataset.

**Figure 14:**
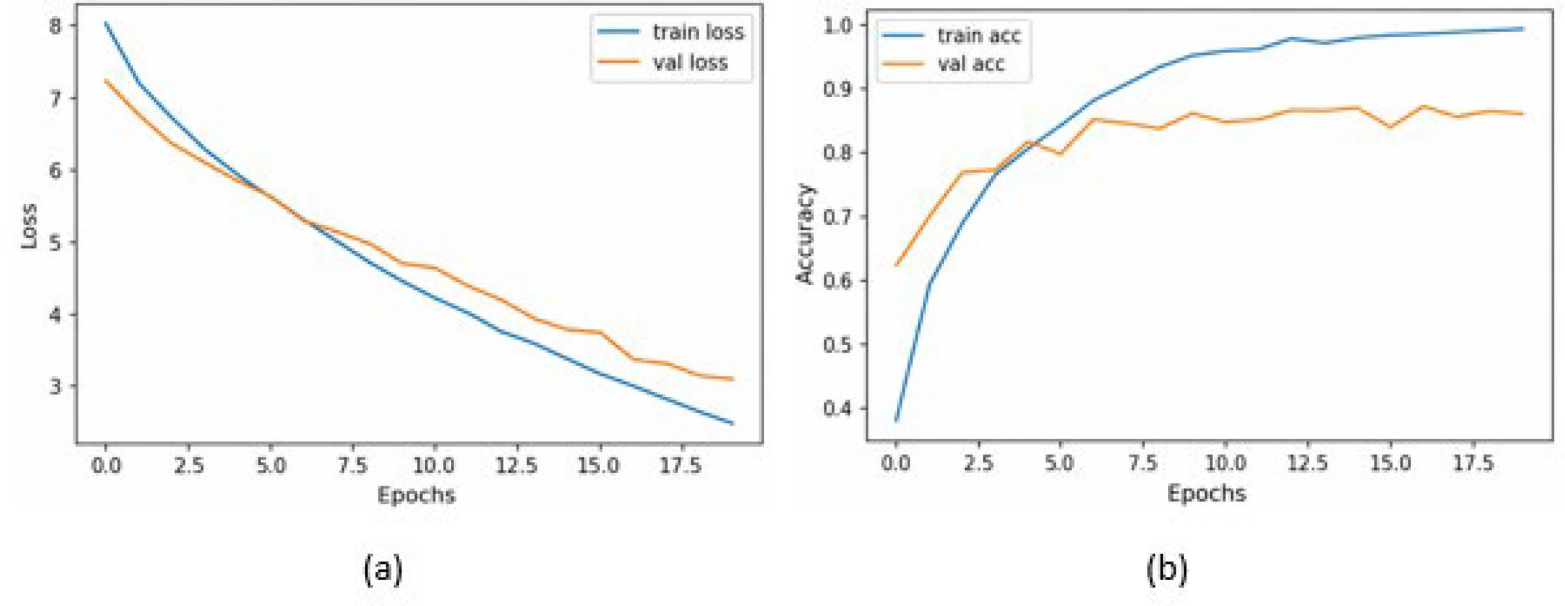
**(a)** Training loss and validation loss vs. number of epochs; **(b)** Training accuracy and validation accuracy vs. the number of epochs.

**Figure 15:**
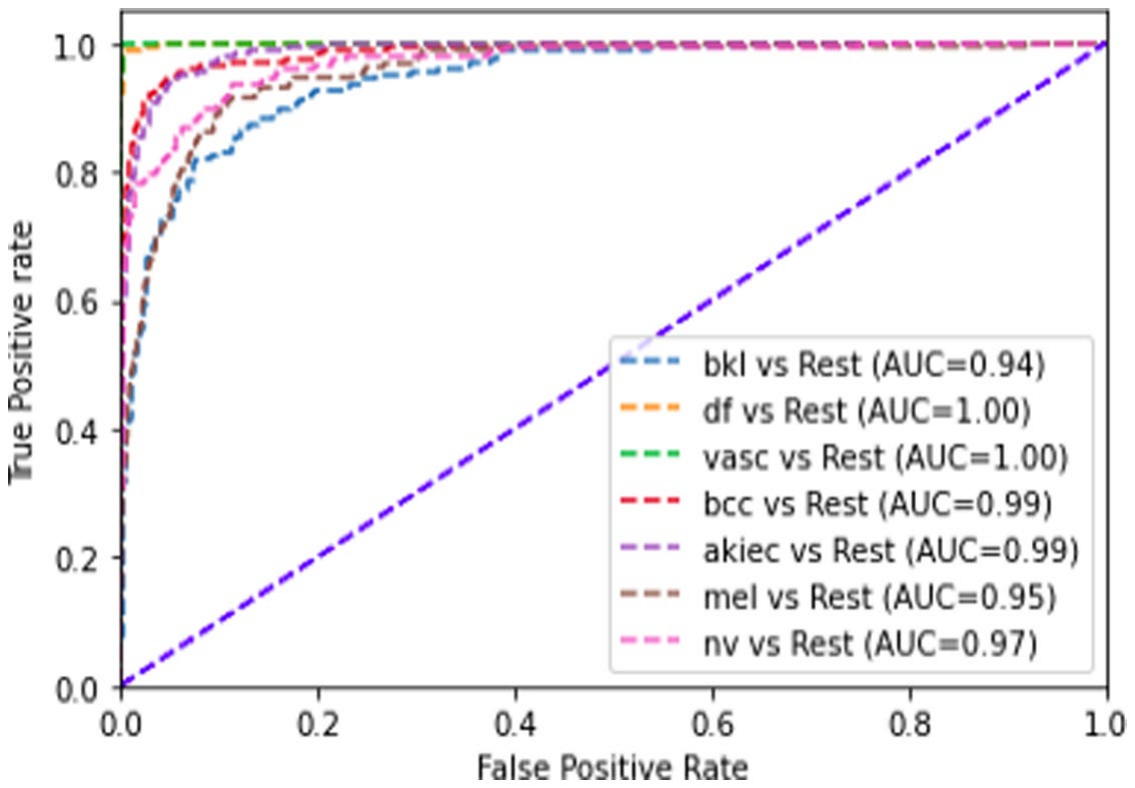
Multiclass ROC curve for HAM10k dataset.

Table 6 presents an overview of the model’s performance on the HAM10k dataset. Figure 13 represents the confusion matrix concerning the same. Figure 14a represents the training loss and validation loss as compared against the number of epochs. Similarly, Figure 14b shows the training and validation accuracy as a function of the number of epochs. Table 7 shows how the model differentiated among the diverse classes in the HAM10k dataset. Figure 15 shows the multiclass ROC curve for the classes in the HAM10k dataset. The AUC value obtained is 0.9744.

**Table 6:** Overall model performance on HAM10k dataset.

Category	Accuracy (%)	Precision (%)	Recall (%)	Specificity (%)	F1-Score (%)
Hold-out cross-validation	86	87	86	85	86
5-Fold cross-validation	85.62	86.64	85.55	85.61	85.07

**Table 7:** Class-wise performance metrics on HAM10k dataset.

Class	Precision	Recall	F1-score	Support	Accuracy
BKL	84	46	59	191	45.55
DF	94	99	96	202	99.00
VASC	100	100	100	210	100
BCC	92	88	90	179	87.70
AKIEC	90	91	91	221	90.95
MEL	63	86	73	184	86.42
NV	81	87	84	213	86.85

### 5.4 Comparison with other research studies

The performance measures obtained from the model’s application to a combination of the HAM10k and PAD-UFES datasets are unique to our proposed study, but to represent the competitiveness of our study, we present a comparison table for studies on the HAM10k and PAD-UFES datasets individually. Table 8 compares the performance of the proposed model on the HAM10k dataset with other existing studies using the same dataset. The proposed model’s performance is noteworthy compared to other studies that employ ML and CNN techniques. Additionally, contrast stretching is comparable to ESRGAN as an image enhancement technique and yields equivalent output accuracy. Table 9 presents the competency of our model in comparison to other existing studies that have been based on the PAD-UFES dataset. Table 9 also presents that our proposed methodology, which uses Vision Transformers and contrast stretching, outperforms diverse implementation techniques, such as CNNs and XGB, as well as novel CNN architectures like HierAttn (Table 10).

**Table 8:** Performance comparison table for the proposed model performance on the HAM10k dataset.

S. No.	Method used	Accuracy (%)
1.	Proposed method	86
2.	Feature, color, and shape extraction using color histogram, Hu moments, and Haralick features (Shetty et al., 2022)	Highest among them, 58.125
3.	Scratch framing of CNN (Nugroho et al., 2019)	78
4.	ESRGAN + ResNet-50 (Alwakid et al., 2022)	86
5.	Collective intelligence system (Popescu et al., 2022)	86.71

**Table 9:** Performance comparison table for the proposed model performance on the PAD-UFES dataset.

S. No.	Method used	Accuracy (%)
1.	Proposed method	92.15
2.	Grand-Cam (Di̇Mi̇Li̇Ler and Sekeroglu, 2022)	86.5
3.	EfficientNetB3 + XGB in Khan et al. (2021)	78
4.	HierAttn (Dai et al., 2022)	91.22
5.	Hyperparametric meta fusion block (Pundhir et al., 2022)	83

**Table 10:** Performance comparison table for the proposed model with other transformer-based dermatology implementations on diverse datasets.

Reference	Method used	Accuracy (%)	Number of diagnostic classes
	Proposed model	93.22	9
Vachmanus et al. (2023)	BEiT with metadata fusion	87.1	8
Nie et al. (2022)	Hybrid CNN (ResNet-50 first 10 layers) + Vision Transformer encoder	89.48	7
Arshed et al. (2023)	Vision Transformer + CNN-based transfer learning	92.14	7
Zhang et al. (2023)	Custom multi-modal transformer with MTP block	77.99	5
Khan and Khan (2023)	VOLO Outlooker block + Transformer block + MLP head	91.02	3

### 5.5 Statistical study

To assess the statistical significance of the proposed model’s performance, a *Z*-score-based hypothesis test was conducted on the per-class accuracies obtained across all three dataset evaluation scenarios: HAM10k (seven classes), PAD-UFES (six classes), and the combined dataset (nine classes).

The *Z*-score was calculated using the standard formula:


$$Z=\frac{\underline{X}-\mu }{\sigma /\sqrt{n}}.$$

where $\underline{X}$ is the observed mean accuracy, *μ* is the baseline (random chance), *σ* is the standard deviation of per-class accuracies, and *n* is the number of classes.

Standard deviation of per-class accuracies:


$$\sigma =\sqrt{\frac{\sum {\left(x_{i}-\underline{x}\right)}^{2}}{n}}=\sqrt{\frac{271.0405\;}{9}}=\sqrt{30.116}=5.488$$

*μ* = 11.11% (random chance, 1/9)

Z-Score computation: Z = $\;\frac{\underline{X}-\mu }{\sigma /\sqrt{n}}\;$ = $\frac{93.37-11.11\;}{5.488/\sqrt{9}}=\frac{\;82.26}{1.829}=44.98$

Standard deviation of per-class accuracies:


$$\sigma =\sqrt{\frac{\sum {\left(x_{i}-\underline{x}\right)}^{2}}{n}}=\sqrt{\frac{382.4269\;}{6}}=7.984$$

*μ* = 16.67% (random chance, 1/6)

*Z*-Score computation:


$$Z=\frac{\underline{X}-\mu }{\sigma /\sqrt{n}}=\frac{91.84-16.67\;}{7.984/\sqrt{6}}=\frac{\;75.17}{3.260}=23.06$$

Standard Deviation of per-class accuracies


$$\sigma =\sqrt{\frac{\sum {\left(x_{i}-\underline{x}\right)}^{2}}{n}}=\sqrt{\frac{2025.1252\;}{7}}=17.009$$

*μ* = 14.29% (random chance, 1/7)

*Z-Score computation: Z =* $\;\frac{\underline{X}-\mu }{\sigma /\sqrt{n}}\;$ *=* $\frac{85.21-14.29\;}{17.009/\sqrt{7}}=\frac{\;70.92}{6.429}=11.03$

Across all evaluated dataset scenarios from Tables 11–13 that are used to find the mean values, the *Z*-scores obtained far exceed the critical thresholds of 1.96 (95% confidence) and 2.58 (99% confidence), confirming that the model’s classification performance is statistically significant well beyond conventional significance levels (*p* ≈ 0). These results confirm that the proposed ViT + MLP architecture yields consistent, robust performance that is not attributable to random variation across both individual and combined diagnostic datasets.

**Table 11:** Mean calculation for the combined dataset (nine classes).

Class	Accuracy (%)	(x − x̄)	(x − x̄)^2^
SCC	86.80	−6.32	39.9424
BCC	87.43	−5.69	32.3761
NEV	94.68	1.56	2.4336
ACK	85.20	−7.92	62.7264
MEL	99.47	6.35	40.3225
SEK	96.61	3.49	12.1801
DF	98.67	5.55	30.8025
BKL	91.47	−1.65	2.7225
VASC	100	6.88	47.3344
Mean (x̄)	93.37	–	271.0405

**Table 12:** Mean calculation for the PAD-UFES dataset (six classes).

Class	Accuracy (%)	(x − x̄)	(x − x̄)^2^
BCC	79.79	−12.05	145.2025
MEL	100	8.16	66.5856
NEV	95.87	4.03	16.2409
SCC	96.23	4.39	19.2721
ACK	81.67	−10.17	103.4289
SEK	97.47	5.63	31.6969
Mean (x̄)	91.84	–	382.4269

**Table 13:** Mean calculation for the HAM10k dataset (six classes).

Class	Accuracy (%)	(x – x̄)	(x – x̄)^2^
BKL	45.55	−39.66	1,572.9156
DF	99.00	13.79	190.1641
VASC	100.00	14.79	218.7441
BCC	87.70	2.49	6.2001
AKIEC	90.95	5.74	32.9476
MEL	86.42	1.21	1.4641
NEV	86.85	1.64	2.6896
Mean (x̄)	85.21	–	2,025.1252

## 6 Conclusion

The proposed study showcases its novelty in representing the implementation of Vision Transformers, contrast stretching, and a lightweight MLP classifier for the purpose of achieving multimodal skin lesion detection and accurate mapping to the corresponding disease class. Additionally, the model’s testing performance was assessed on a robust dataset combining the clinically curated HAM10k and a relatively general PAD-UFES dataset, thereby providing an exclusive, extensive, and exhaustive nine-class multinomial classification. As stated in Section 5.1, the proposed model achieves a testing accuracy of 93.22% and a training accuracy of 98%, which is a great milestone given the number of classes considered for classification. The model implementation is lightweight and therefore has the potential to be implemented on mobile and resource-constrained devices. Authors would therefore like to convey that the proposed study is intended to serve as a first-touch screening system and is not intended to replace clinical diagnosis. As part of future enhancements, the proposed study can be trained and modified to cover a greater number of diagnostic classes within the classification scope. In addition, we can also make the model in the binary classification mode to distinguish between malignant and benign lesions.

## Data Availability

Publicly available datasets were analyzed in this study. This data can be found at: the datasets generated during and/or analyzed during the current study are available in the following databases 1. HAM10000 dataset, https://www.kaggle.com/datasets/kmader/skin-cancer-mnist-ham10000; 2. PAD-UFES dataset, https://data.mendeley.com/datasets/zr7vgbcyr2/1.
